# P-1911. Outcomes of a Statewide Educational Intervention Focused on Reducing COVID-19 Health Disparities Through a Quality Improvement Approach

**DOI:** 10.1093/ofid/ofaf695.2080

**Published:** 2026-01-11

**Authors:** Priscila R Armijo, M Salman Ashraf, Adati Tarfa, Elizabeth Lyden, Jasmine R Marcelin, Jonathan H Ryder, Jeffrey Wetherhold, Mahelet Kebede, Subhadra Mandadi, Precious S Davis, Shirley F Delair, Kelly Cawcutt, Andrea D Jones, Mahliqha Qasimyar, Gale M Etherton, Erica Stohs, Nada Fadul

**Affiliations:** University of Nebraska Medical Center, Omaha, Nebraska; University of Nebraska Medical Center, Omaha, Nebraska; Yalyalee School of Medicine, New Haven, Connecticut; University of Nebraska Medical Center, Omaha, Nebraska; University of Nebraska Medical Center, Omaha, Nebraska; University of Nebraska Medical Center, Omaha, Nebraska; Ohia Advisors, Boston, Massachusetts; isat advisors, Washington, District of Columbia; University of Nebraska Medical Center, Omaha, Nebraska; University of Nebraska Medical Center, Omaha, Nebraska; University of Nebraska Medical Center, Omaha, Nebraska; University of Nebraska Medical Center, Omaha, Nebraska; University of Nebraska Medical Center, Omaha, Nebraska; University of Nebraska Medical Center, Omaha, Nebraska; University of Nebraska Medical Center, Omaha, Nebraska; Creighton University Medical Center, Omaha, NE; University of Nebraska Medical Center, Omaha, Nebraska

## Abstract

**Background:**

The COVID-19 Disparities ECHO Project sought to enhance healthcare and public health professionals’ knowledge in four domains – health equity, cultural sensitivity, infection prevention and control, and quality improvement (QI) – and support implementation of QI initiatives promoting equity in clinical environments. We evaluated the project’s outcomes, including reach, effectiveness, adoption, and implementation.

**Figure:**
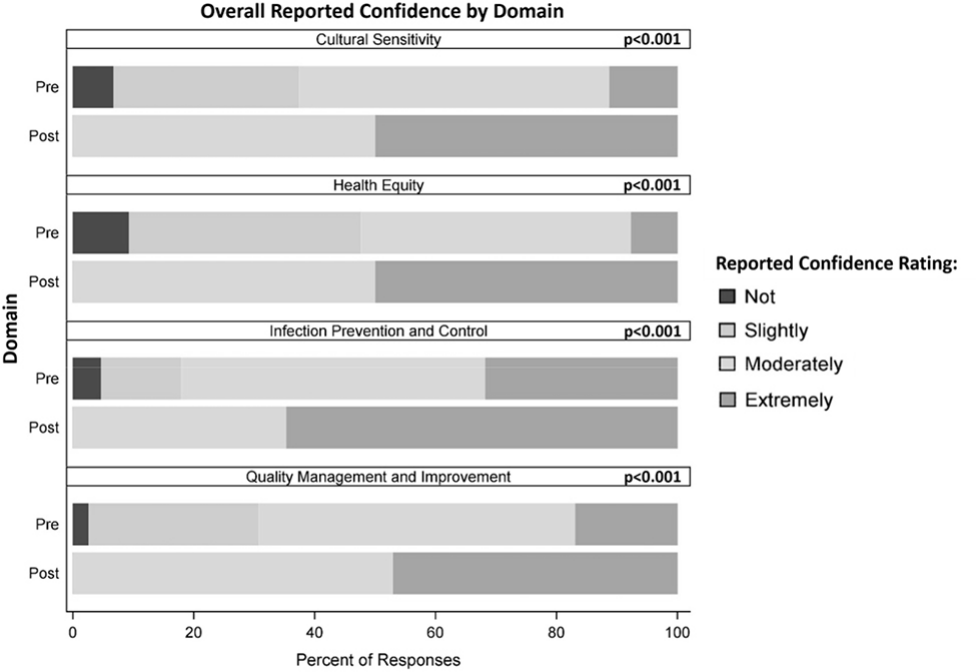

**Figure:**
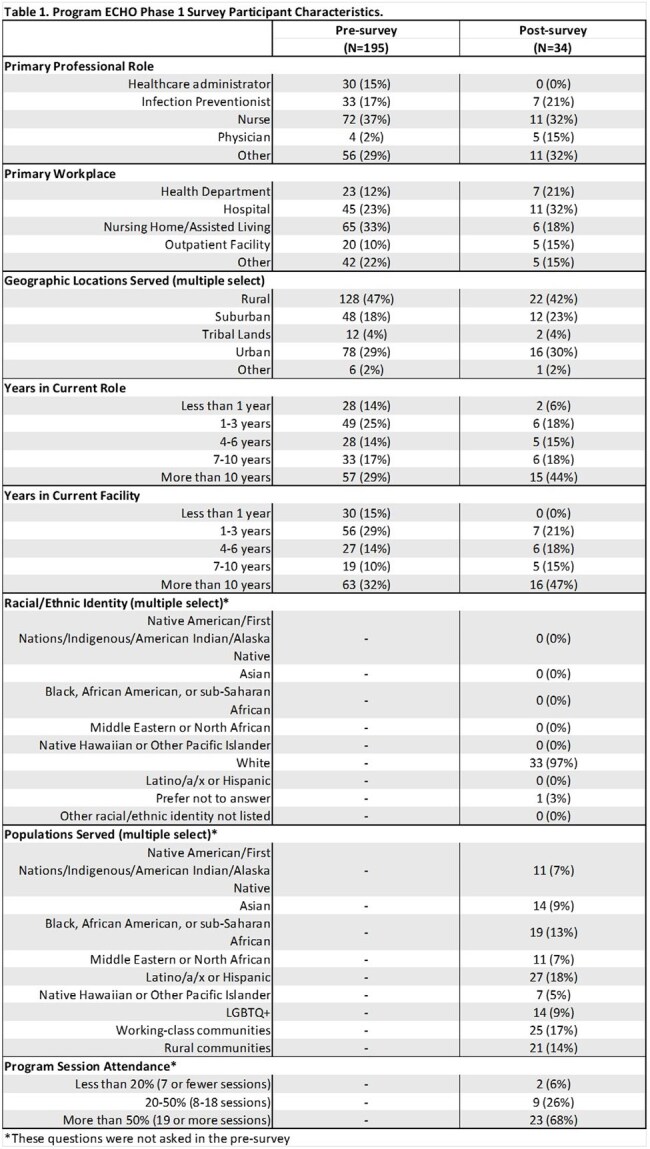

**Methods:**

Virtual, synchronous semi-monthly 90-minute didactic sessions (Phase 1, Nov/21–May/23) followed by monthly QI-focused sessions (Phase 2, Jun/23–May/24) were held. In-session polls and anonymous pre- and post-phase 1 survey results, including demographics, attendance, confidence in each domain, content relevance, session experience, evaluation, and number of QI projects were analyzed using descriptive and comparative statistics.

**Figure:**
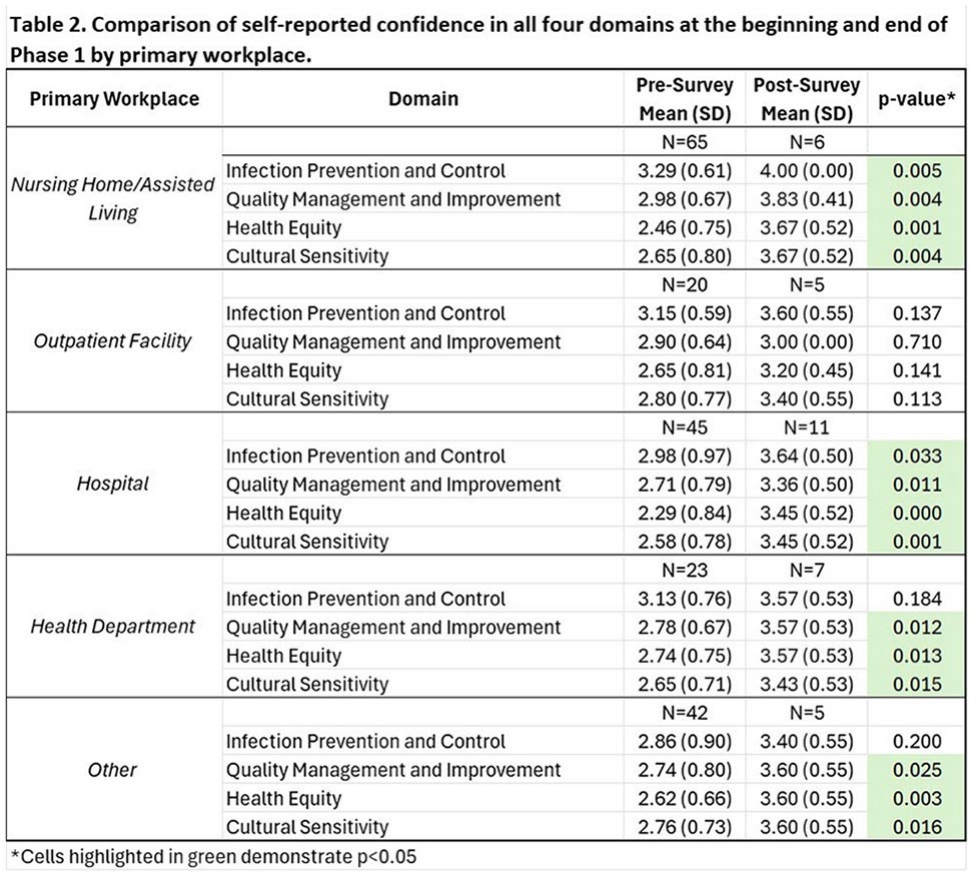

**Figure:**
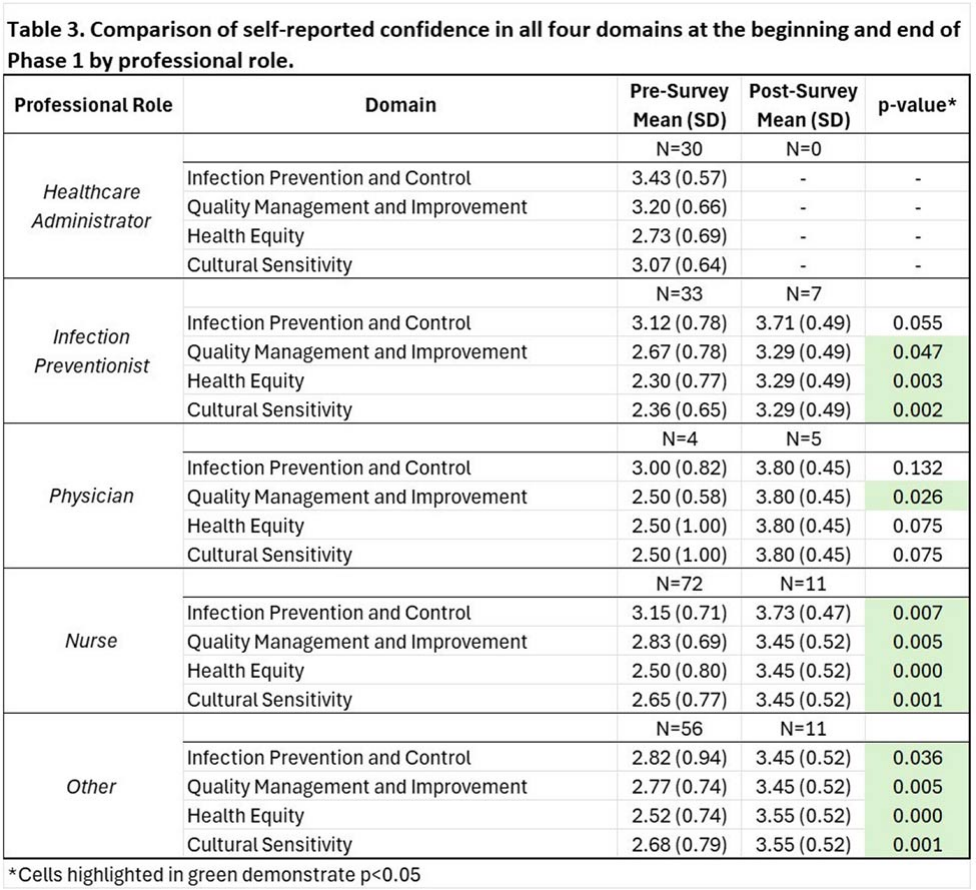

**Results:**

Project ECHO had 510 unique participants (420 in Phase 1, 197 in Phase 2). Response rates for the pre- and post-surveys in Phase 1 were 46.4% (N=195) and 8.1% (N=34), respectively (Table 1). There was a statistically significant increase in overall reported confidence between the pre- and post-surveys across all four domains (all p< 0.001, Figure 1). Self-reported confidence significantly increased across all workplace settings except outpatient settings (Table 2) and all professions except physicians (Table3). During session polls, majority of attendees reported that they had learned something new in the previous session (average=58%), and they could apply the content from that session in their work (average=93%). At the post-survey, 69% reported attending more than 50% of sessions, 63% reported that content learned will definitely be used in their work, and 66% that it changed how they work with patients. The Net Promoter Score was 63%, suggesting high participant satisfaction. Eighteen QI projects were initiated (11 in Phase 1, 7 in Phase 2).

**Conclusion:**

Project ECHO showed promise in improving healthcare/public health professionals’ awareness and cultural sensitivity in healthcare settings. Despite the overall significant increase in confidence, variation existed across different roles and workplace settings. Future studies should evaluate the long-term sustainability of this project.

**Disclosures:**

M. Salman Ashraf, MBBS, Merck & Co. Inc: Grant/Research Support Erica Stohs, MD MPH, bioMerieux, Inc.: Grant/Research Support|Merck Co, Inc: Grant/Research Support Nada Fadul, MD, ViiV Healthcare: Advisor/Consultant|ViiV Healthcare: Grant/Research Support

